# The secreted autotransporter toxin (Sat) does not act as a virulence factor in the probiotic *Escherichia coli* strain Nissle 1917

**DOI:** 10.1186/s12866-015-0591-5

**Published:** 2015-10-30

**Authors:** Lorena Toloza, Rosa Giménez, María Jose Fábrega, Carina Shianya Alvarez, Laura Aguilera, María Alexandra Cañas, Raquel Martín-Venegas, Josefa Badia, Laura Baldomà

**Affiliations:** Departament de Bioquímica i Biología Molecular, Institut de Biomedicina de la Universitat de Barcelona, Facultat de Farmàcia, Universitat de Barcelona, E-08028 Barcelona, Spain; Departament de Fisiologia, Facultat de Farmàcia, Universitat de Barcelona, E-08028 Barcelona, Spain

**Keywords:** *Escherichia coli* Nissle 1917, Probiotic, Secreted autotransporter toxin (Sat), Serine protease autotransporters of *Enterobacteriaceae* (SPATEs)

## Abstract

**Background:**

*Escherichia coli* Nissle 1917 (EcN) is a probiotic used in the treatment of intestinal diseases. Although it is considered safe, EcN is closely related to the uropathogenic *E. coli* strain CFT073 and contains many of its predicted virulence elements. Thus, it is relevant to assess whether virulence-associated genes are functional in EcN. One of these genes encodes the secreted autotransporter toxin (Sat), a member of the serine protease autotransporters of *Enterobacteriaceae* (SPATEs) that are secreted following the type V autotransporter pathway. Sat is highly prevalent in certain *E. coli* pathogenic groups responsible for urinary and intestinal infections. In these pathogens Sat promotes cytotoxic effects in several lines of undifferentiated epithelial cells, but not in differentiated Caco-2 cells.

**Results:**

Here we provide evidence that *sat* is expressed by EcN during the colonization of mouse intestine. The EcN protein is secreted as an active serine protease, with its 107 kDa-passenger domain released into the medium as a soluble protein. Expression of recombinant EcN Sat protein in strain HB101 increases paracellular permeability to mannitol in polarized Caco-2 monolayers. This effect, also reported for the Sat protein of diffusely adherent *E. coli*, is not observed when this protein is expressed in the EcN background. In addition, we show that EcN supernatants confer protection against Sat-mediated effects on paracellular permeability, thus indicating that other secreted EcN factors are able to prevent barrier disruption caused by pathogen-related factors. Sat is not required for intestinal colonization, but the EcN*sat::cat* mutant outcompetes wild-type EcN in the streptomycin-treated mouse model. Analysis of the presence of *sat* in 29 strains of the ECOR collection isolated from stools of healthy humans shows 34.8 % positives, with high prevalence of strains of the phylogenetic groups D and B2, related with extra-intestinal infections.

**Conclusions:**

Sat does not act as a virulence factor in EcN. The role of Sat in intestinal pathogenesis relies on other genetic determinants responsible for the bacterial pathotype.

**Electronic supplementary material:**

The online version of this article (doi:10.1186/s12866-015-0591-5) contains supplementary material, which is available to authorized users.

## Background

*Escherichia coli* Nissle 1917 (EcN) (serotype O6:K5:H1) is a Gram-negative probiotic used in the treatment of intestinal disorders, especially inflammatory bowel diseases [1–3]. This strain, originally isolated from a soldier who survived a severe outbreak of diarrhoea during the First World War, is a good colonizer of the human gut and positively affects gastrointestinal homeostasis and microbiota balance. EcN modulates the expression of antimicrobial peptides, increases secretion of IgA and mucin, and promotes anti-inflammatory modulation of the immune response [4]. In addition, this probiotic modulates the intestinal epithelial barrier through upregulation and redistribution of tight junction proteins [5–7].

The EcN genome has been sequenced (genome size 5,441,200 bp) and is predicted to contain 5,324 coding sequences, among which 190 genes are strain-specific [8, 9]. EcN is furnished with a large repertoire of fitness factors that promote its competitiveness, which probably explains its success as a probiotic. Among these fitness factors there are microcins, iron uptake systems, adhesins and proteases that contribute to the colonization of the human gut [10–12]. Genes encoding these factors are mainly clustered in genomic islands and some smaller groups. Comparative genomic hybridization studies and genome sequencing revealed that the probiotic strain EcN is closely related to the uropathogenic *E. coli* strain (UPEC) CFT073 [9, 12]. Even though the two strains use different communication strategies with the host, their gene profiles are very similar (differing only in a few hundred genes). EcN lacks genes encoding defined virulence factors such as haemolysin and P-fimbrial adhesin, but most of the predicted CFT073 virulence elements are also present in the EcN genome. A transcriptomic analysis revealed that many UPEC virulence-related genes are expressed in the probiotic EcN [13]. The borderline between virulence and fitness factor is in some cases diffuse, as virulence depends on factors that increase fitness during colonization of specific host niches. One of these factors is the secreted autotransporter toxin Sat, encoded in EcN by a gene located in the genomic island II. Sat belongs to the subfamily of serine protease autotransporters of *Enterobacteriaceae* (SPATEs). This family is composed of extracellular proteases with diverse functions, normally associated with virulence of Gram-negative pathogens, which are secreted by the type Va secretion pathway [14, 15]. These proteins display the typical features of autotransporters: an N-terminal signal sequence, a passenger domain secreted into the extracellular medium and a C-terminal β-barrel domain involved in protein translocation through the outer membrane. Proteins of the SPATE family have been classified into two main groups according to their structure and activity as well as to phylogenetic criteria. Class I includes proteins with cytotoxic activity and class II comprises non-cytotoxic proteins with roles in colonization and immunomodulation [16, 17].

Sat is a class I protease synthesized as a precursor (142 kDa) and processed at the N-terminal end on its way to the periplasmic space via the Sec secretion system (residues 1–49). This protein is subsequently self-transported through the outer membrane and undergoes another proteolytic cleavage which releases the protease domain (residues 50–1018; 107 kDa) and keeps the C-terminal domain anchored to the outer membrane (residues 1019–1295; 31 kDa) [18]. Sat function has been studied in pathogens, specifically in the UPEC strain CFT073, and in Afa/Dr difussely adherent *E. coli* (DAEC) responsible for urinary tract and intestinal infections. *In vitro* studies showed that Sat from UPEC strains displays proteolytic activity on casein, spectrin, fodrin and coagulation factor V. Mucin, pepsin or IgA were not degraded by Sat [18–20]. In several cellular models of kidney, bladder and undifferentiated epithelial cells Sat promotes cytotoxic effects including vacuolization, autophagy and cell detachment [18, 21, 22]. Studies using subclonfluent HeLa cells exposed to Sat from Afa/Dr DAEC strains revealed that these cytotoxic effects are preceded by F-actin cytoskeleton disassembly [22]. Moreover, several reports provide evidence that Sat is internalized by host cells and localized both in the cytosol and in nucleus [20, 22]. In differentiated Caco-2 cells, this toxin induces rearrangements of the tight junctions-associated proteins ZO-1 and ZO-2 and occludins, leading to an increase in both fluid dome formation and paracellular permeability to small molecules like mannitol, without altering the monolayer transepithelial electrical resistance (TER). In this model, Sat of DAEC does not modify the paracellular permeability to fluorescein-5-sulphonic acid (478 Da), nor does it trigger dissociation of the F-actin network [23]. No experimental evidence of Sat-mediated proteolysis of tight junction-proteins has been reported to date. *In vivo* experiments carried out with Sat from a DAEC strain isolated from a child with diarrhea showed that this toxin induces fluid accumulation and histological effects in rabbit ileon loops. However, the intensity of the observed effects displayed great variation between animals, suggesting that other host factors may influence the establishment of diarrheal diseases [24].

As EcN is considered a safe probiotic [4, 25] it is relevant to test whether virulence-associated genes are expressed and whether the encoded proteins are functional. We report here the expression of *sat* during the colonization of the mouse intestine by EcN, and provide evidence that EcN Sat is a functional protease. We report that the negative effects of Sat on the paracellular permeability of Caco-2 monolayers *in vitro* are not seen in the probiotic strain. In addition, we have performed colonization experiments using the streptomycin-treated mouse model, as well as a PCR survey to evaluate prevalence of *sat* among the *E. coli* reference collection (ECOR) strains from human intestinal origin. Our results indicate that the putative pathogenic role of Sat in the intestinal tract depends on the expression of other strain virulence determinants.

## Methods

### Bacterial strains and growth conditions

*E. coli* strains and plasmids used in this study are listed in Table 1. Bacterial cells were routinely grown at 37 °C in Luria-Bertani broth (LB). When indicated, cells were grown in minimal medium (MM) [26] supplemented with 0.2 % glucose and 0.02 % casamino acids [13] or DMEM (Dulbecco’s Modified Eagle Medium). Growth was monitored by measuring the optical density at 600 nm (OD_600_). Bacterial cells were counted by platting serial dilutions on LB-plates containing the appropriate antibiotic. When required, ampicillin (Ap) (100 μg/ml), chloramphenicol (Cm) (20 μg/ml), streptomycin (Sm) (100 μg/ml), rifampicin (Rf) (25 μg/ml) or tetracycline (Tc) (12.5 μg/ml) was added to the medium. Expression of β-galactosidase was achieved by addition of 5 mM isopropyl-β-D-1-thiogalactopyranoside (IPTG) to the culture medium.

**Table 1:** Strains and plasmids used in this study

Strain or plasmid	Genotype or description	Source or reference
*E. coli* strains		
XL1Blue	*recA1 lac endA1 gyrA96 thi hsdR17 supE44 relA1* (F’ *proAB lacI* ^*q*^ *lacZ* ΔM15 Tn*10*)	Stratagene
DH5α	F^−^ *Φ80lacZΔM15 Δ(lacZYA-argF) U169 recA1 endA1 hsdR17*(r_k_^−^ m_k_^−^) *phoA supE44* λ^*−*^ *thi- gyrA96 relA1*	Gibco BRL
HB101	F^−^ *mcrB mrr hsdS20*(r_B_^−^ m_B_^−^) *recA13 leuB6 ara-14 proA2 lacY1 galK2 xyl-5 mtl-1* rpsL20(Sm^R^) glnV44 λ^−^	ATCC 33694
S17(λpir)	Tp^r^ Sm^r^ *recA thi pro hsdR hsdM* ^*+*^RP4::2-Tc::Mu::Km Tn7 λ	Biomedal
Nissle 1917 (EcN)	Non-pathogenic probiotic isolate (O6:K5:H1)	Ardeypharm
EcN*sat::cat*	Nissle 1917 *sat*::*cat*; Cm^r^	This study
E2348/69	Wild type EPEC O127:H6 Sm^r^	B.B. Finlay
ECOR collection strains	Human stool isolates	[43]
Plasmids		
pBR322	Vector for cloning, Ap^r^ Tc^r^	Biolabs
pSat	Gene *sat* from EcN cloned in pBR322, Ap^r^	This study
pSat-S256I	Gene *sat* from EcN encoding the mutated Sat-S256I cloned in pBR322, Ap^r^	This study
pCAT19	Source of *cat* gene, Ap^r^ Cm^r^	[26]
pUT mini-Tn*5* Tc	tnp* gene of Tn*5*-IS*50*R inserted in SalI site of pGP704; mini-Tn*5* Tc transposable element, Ap^r^ Tc^r^	Biomedal
pFU34	Plasmid for transcriptional fusions to the reporter gene *gfp*_*mut3.1*_, Ap^r^	[28]
pFU34-sat	Promoter fusion Φ(*sat-gfp*_*mut3.1*_) in PFU34, Ap^r^	This study

### Construction of mutant strains

Rf- or Sm-resistant mutants were obtained by spontaneous mutation. To construct an EcN isogenic *sat* mutant, the 3 Kb-fragment encoding the Sat passenger domain was amplified with primers Mut-sat-Fw ACAGGATCCGCAAATATTGATATATATCAAATGTATGG) and Mut-sat-Rev (ACAGAATTCGTTGACCTCAGCAAGGAAG) and cloned into the EcoRI/BamHI restriction sites of plasmid pUC18Not (Biomedal). The resulting recombinant plasmid was then digested with SwaI (with a single restriction site in the *sat* gene) and ligated with a *cat* cassette that confers resistance to Cm. This cassette was obtained by digestion of plasmid pCAT19 [27] with SmaI. The recombinant plasmid was then digested with NotI and the fragment *sat::cat* was cloned into the NotI restriction site of vector pUTmini-Tn5 Tc (Biomedal). After electroporation of *E. coli* S17 (λpir) with this final construct, the introduction of the *sat::cat* mutation into the EcN chromosome was performed by conjugation using as a recipient strain a Rf-resistant derivative of EcN. Transconjugants having the Rf^r^ Cm^r^ Tc^s^ Ap^s^ phenotypes were selected. Chromosomal insertion was confirmed by PCR. This insertional inactivation did not cause polarity on downstream genes as *sat* is transcribed as a single unit. Growth rates of the *sat* mutant strain in LB or DMEM were indistinguishable from that of the parental EcN strain.

### DNA manipulation and site-directed mutagenesis

Bacterial genomic DNA was obtained using the Wizard Genomic DNA purification kit (Promega), and plasmid DNA was prepared using the Wizard Plus SV Midipreps DNA purification system (Promega). DNA manipulations were performed essentially as described elsewhere [28]. DNA fragments were amplified by PCR using *E. coli* chromosomal DNA as a template. PCR reactions were performed with Taq DNA polymerase or *pfu* DNA polymerase in standard conditions. DNA sequencing was carried out with an automated ABI 377 DNA sequencer and fluorescent dye termination methods.

The *sat* gene from EcN was amplified by PCR using primers pBR-sat-Fw (TACGGATCCGGATCAGGGTTGGCAATATCG) and pBR-sat-Rev (GCTAATAATGAGAGCAAGAGCGAT) and cloned into pBR322 between BamHI and NruI restriction sites. This construct encompasses the complete *sat* coding sequence and the 435 bp-region upstream the ATG start codon. Site-directed mutagenesis of the Sat catalytic serine residue (S256) was performed using the Quick change site-directed mutagenesis kit (Stratagen) following the manufacturer’s instructions. The set of primers used to replace this residue to isoleucine (S256I) were TCGGAGACATCGGCTCTGG and CCAGAGCCGATGTCTCCGA.

### Construction of *sat-gfp* promoter fusion and detection of the reporter protein

Plasmid pFU34-sat (*sat-gfp* promoter fusion) was constructed by amplification of the *sat* upstream region with primers Sat-gfp-Fw (GGCGGATCCCGGTCTGAATAACGCAGCTAG) and Sat-gfp-Rev (GCACGTGTCGACATTCATATATTCTCTCAACTCATTTATTGAATGAACA) from EcN genomic DNA and cloned into the BamHI/SalI sites of plasmid pFU34 [29]. GFP expressed from EcN cells harboring plasmid PFU34-sat was visualized with a fluorescence microscopy (Leica D1000). For the analysis of the reporter gene expression under *in vitro* conditions, EcN harboring pFU34-sat or pFU34 as a control was diluted 1:50 in fresh culture medium from overnight cultures and grown at 37 °C to exponential phase. Fluorescence emitted by bacterial cells was measured in a microtiter plate reader (Turner BioSystems Modulus™ II Microplate). The data obtained from three independent cultures were given as relative fluorescence units per OD_600_ (RLU/OD).

### Isolation of secreted proteins

Isolation of secreted proteins was performed as previously described [30]. Briefly, overnight cultures in LB were diluted 1:50 in the indicated culture media and incubated at 37 °C. After 8 h of growth (OD_600_ of 1.0) bacteria were collected by centrifugation (5,000 x *g*, 10 min, 4 °C), and the supernatant was passed through a 0.22 μm-pore-size filter (Millipore). For protease activity assays, cell-free supernatants were concentrated 500-fold using Centricon-Plus-70 centrifugal filters (Millipore) with a molecular weight cut-off of 100 kDa. Protein concentration was quantified by the method of Lowry [31]. For Western blot analysis, the proteins in the filtrate were precipitated by incubation on ice for at least 1 h with 10 % trichloroacetic acid (TCA). The protein pellet was washed in 90 % (v/v) ice-cold acetone, air-dried and suspended in loading buffer before being resolved by sodium dodecyl sulphate (SDS)- polyacrylamide gel electrophoresis (PAGE) [32]. Outer membrane vesicles (OMVs) were isolated from cell-free supernatants as described previously [33].

### Serine protease and spectrin cleavage assays

Serine protease activity was measured using the p-nitroanilide substrate assay [20]. Concentrated supernatant (200 μg protein) was added to a reaction mixture in 0.1 M morpholinepropanesulfonic acid buffer pH 7.3, containing 200 mM NaCl, 0.01 mM ZnSO_4_ and 1 mM methoxysuccinyl-Ala-Ala-Pro-Val p-nitroanilide as the substrate (Sigma-Aldrich). Samples were incubated at 37 °C during 18 h and substrate hydrolysis was monitored at 505 nm. As a control, protease activity was also assayed after preincubation of samples for 30 min at 37 °C with 1 mM phenylmethanesulfonyl fluoride (PMSF). The spectrin cleavage assay was performed as described elsewhere [20]. Reaction mixtures (20 μl) containing spectrin (4 μg) (Sigma-Aldrich) and concentrated culture supernatants were incubated at 37 °C for 24 h. Reaction products were resolved by SDS-PAGE.

### Western blot analysis

For Western blot analysis, protein samples were separated on 10 % SDS-PAGE and transferred to a HyBond-P polyvinylidene difluoride membrane by using a Bio-Rad MiniTransblot apparatus. The membrane was blocked in PBS-0.05 % Tween-20 and 5 % skimmed milk (blocking solution) for 1 h at room temperature, and then incubated with specific antibodies against Sat (1:5,000 dilution in blocking solution) or with antibodies against β-galactosidase (Abcam, 1:10,000 dilution) for 16 h at 4 °C. Anti-Sat polyclonal antibodies (rabbit) were raised against a conjugated peptide (CKSNNQQTSFDQPDW) of the Sat passenger domain selected by its antigenicity and surface exposure features prediction (Genscript). The secondary antibody was donkey anti-rabbit immunoglobulin horseradish peroxidase-linked, diluted 1:15,000 in blocking solution. The protein-antibody complex was visualized by using the ECL Plus Western blotting detection system (Amersham Pharmacia Biotech).

### Cell culture and infection conditions

HeLa or Caco-2 human colonic adenocarcinoma cells (ATCC HTB-37) were routinely grown in DMEM containing 25 mM HEPES, non-essential amino acids, 100 U/ml of penicillin G and 100 μg/ml of streptomycin. The medium was supplemented with 10 % (v/v) of heat inactivated fetal calf serum (FCS) (Gibco BRL, MD, USA). Cells were incubated at 37 °C in 95 % air / 5 % CO_2_.

For permeability assays, Caco-2 cells were seeded on polycarbonate filter supports (0.4 μm, Transwell Corning) and experiments were performed when confluent monolayers were fully polarized (15–18 days postconfluence). For infection studies, *E. coli* cells were grown in LB overnight at 37 °C; the culture was then diluted 1:50 with fresh medium and grown until the OD_600_ reached 0.5-1.0. Bacterial cells were collected by centrifugation and resuspended in DMEM plus 1 % mannose. Two hours before infection, monolayers of Caco-2 cells were washed twice in phosphate-buffered saline (PBS; 140 mM NaCl, 2.7 mM KCl, 10 mM Na_2_HPO_4_, 1.8 mM KH_2_PO_4_, pH 7.3) and shifted to serum and antibiotic-free medium. Caco-2 cells were treated with bacterial suspensions from the apical side for 3 h at a multiplicity of infection (MOI) of 100. When indicated, bacterial concentrated supernatants (200 μg protein) where added to the apical compartment and incubation was extended for 3 h.

### Transepithelial resistance and permeability measurement

After apical treatment, transepithelial resistance (TER) was determined with a Millicel-ERS-2 volt-ohmmeter (Millipore, Bedford, MA). The background of the supporting membrane in filters was subtracted from all readings before calculations. Results were expressed as Ω · cm^2^ monolayer surface area.

Permeability of Caco-2 cell monolayers was determined by measuring the paracellular passage of [^3^H]-D-mannitol from apical to basolateral compartments of the chamber culture (transwell culture plate inserts; 0.4 μm) as described elsewhere [34]. Monolayers were washed with modified Krebs buffer (137 mM NaCl, 5.4 mM KCl, 2.8 mM CaCl_2_, 1.0 mM MgSO_4_, 0.3 mM NaH_2_PO_4_, 10 mM D-glucose and 10 mM HEPES/Tris, pH 7.4), and placed in culture wells containing 1.5 and 0.5 ml of modified Krebs buffer in the basolateral and apical compartments, respectively. Apical medium contained 0.2 mCi/mL D-[2-^3^H]mannitol. Cells were incubated for 15 min at 37 °C. After this time, the basolateral medium was withdrawn and radioactivity was counted in a scintillation counter (1500 Tri-Carb®, Packard, Downers Grove, IL). Results were expressed as the percentage of recovery relative to total radioactivity.

### Cytotoxicity assays

EcN Sat cytotoxicity was determined by the 3-(4,5-dimethylthiazol-2-yl)-2-5-diphenyltetrazolium bromide (MTT, Sigma-Aldrich) assay in HeLa cells as described elsewhere [22]. HeLa cells were seeded into 96-well plates at 1 x 10^4^ cells/well and grown to 90 % confluency. Before use, cells were kept overnight with medium containing 0.05 % FCS. Cells were washed twice with PBS and exposed to cell-free culture supernatants in the presence of fresh media deprived of FCS. At the indicated times, cell viability was determined the addition of MTT (1 mg/ml final concentration) followed by 1 hour- incubation at 37 °C. After solubilization with DMSO the plates were read at 570 nm using a Modulus™ Microplate Photometer (Turner BiSystems). The results were expressed as percentage of cell survival relative to the control (untreated cells).

### Mouse colonization experiments

The streptomycin-treated mouse model, which overcomes colonization resistance in conventional animals, was used here to study the competition in gut colonization between Sm-resistant EcN and the isogenic EcN*sat::cat* mutant. The model was adapted from previous reports [35, 36].

The strains used in this study were spontaneous Sm^r^ mutants, displaying resistance to antibiotic concentrations greater than 2 mg/ml. Both strains EcN Sm^r^ and EcN*sat::cat* Sm^r^ display the same point mutation in *rpsL*, resulting in the replacement of the lysine residue at position 43 by an isoleucine (AAA has changed to ATA). Therefore differences in the colonization abilities between these strains could not be attributed to different antibiotic resistance degrees. In addition both strains were resistant to Rf, and EcN*sat::cat* was resistant to Cm. Four male CD-1 mice (eight- to ten-weeks old) were provided with drinking water *ad libitum* containing streptomycin sulfate (5 g/l) from 24 hours prior to inoculation and over the entire course of the experiment. Stool samples were proved to be free of Sm-resistant bacteria before inoculation. Bacterial strains, were grown overnight in LB, diluted 1:200 and incubated to exponential phase (OD_600nm_ of 0.5). Then, bacterial cells were collected by centrifugation, washed and resuspended in PBS to a final concentration of 1x10^6^ CFU/ml. A mixed suspension (1:1) of strain EcN Sm^r^ and the isogenic *sat::cat* mutant was prepared to a final concentration of 1x10^6^ CFU/ml, and 0.2 ml of this inoculum was administrated orogastrically (total number of bacteria 2x10^5^). At the indicated times over 18 days post-infection, mice were transferred individually to clean cages and fresh fecal samples were collected. Feces were weighed, diluted and homogenized in sterile PBS. Serial dilutions were plated on LB-agar containing Sm (100 μg/ml) and Rf (25 μg/ml). Plates were incubated for 16 to 20 h at 37 °C before quantification of bacteria by plate counts. To distinguish the two strains, 100 colonies from these plates were screened onto LB-plates containing Sm (100 μg/ml) and Cm (20 μg/ml). Results were expressed as CFU/g of feces. Three independent colonization experiments were performed, with identical results. Pooled data from the three independent biological replicates were presented. Colonization experiments with single bacterial strains were performed in parallel (*n* = 4, two independent biological assays).

To isolate and enumerate bacteria adhered to the mucus layer, on day 18 post-infection mice were sacrificed. Sections of 4 to 5 cm of the ileum (at 1 cm from the cecum) and the ascendant colon were removed from each mouse. Each section of the intestine was washed three times with PBS to expel the luminal content. Then, the mucus layer from each fragment of intestine was scraped, transferred to a sterile tube, weighed and suspended into 1 ml of PBS. Samples were homogenized by vortexing and plated on LB-agar in the presence of Sm and Rf. After colony counting, the proportion of each strain was evaluated by picking 100 colonies on LB-plates containing Cm as described above. Bacteria were quantified as CFU/g of mucus.

The colonization experiments for *in vivo* analysis of *sat* expression were performed using essentially the same mouse model, but adding Ap (2 g/l) instead of Sm to the drinking water. Briefly, two male CD-1 mice (eight- to ten-weeks old) were orogastrically inoculated with 0.2 ml of a suspension of EcN cells harboring plasmid PFU34-sat (2x10^5^CFU) or PFU34 (2x10^5^CFU) as a control. Five days post-infection, fresh fecal samples, as well as the mucus layer from the ileon and colon sections, were collected and processed for quantification of bacteria as described above, using in this case LB-plates containing Ap (100 μg/ml). A drop of each fecal or mucosal suspension was deposited on a slide and visualized with a fluorescence microscopy (Leica D1000).

### Flow cytometry

Human peripheral blood mononuclear cells (PBMCs) were isolated from fresh buffy coats of healthy human donors by using Histopaque^R^-1077 (Sigma-Aldrich) following the manufacturer instructions. Buffy coats were provided by the “Banc de Sang i Teixits” of Barcelona according to the signed agreement with the Institution. Cells were cultured in DMEM containing gentamicin. PBMCs were left untreated or incubated with bacterial concentrated culture supernatants (200 μg total protein) for 3 h. For antibody staining we followed the manufacturer’s recommended protocol. Briefly, PBMCs (1x10^6^ cells/ml; 100 μl) were blocked in PBS + 5 % bovine serum albumin + 0.1 % NaN_3_ at 4 °C. After washing, cells were incubated with the primary antibody anti-LFA-1 (anti-CD11 + CD18, Abcam) (1:100 dilution in blocking solution) at 4 °C for 1 h, followed by 30 min-incubation with the secondary antibody Alexa Fluor 488 goat anti-mouse IgG (Life Technologies). Cell viability was assessed by propidium iodide staining. Data were analysed using the M X P Cytometry software (Cytometer FC500) in the Scientific and Technological Centres of the University of Barcelona (CCiT-UB).

### Statistical analysis

Results were presented as mean ± standard error (SE). Data analysis was performed using SPSS Statistics 20 package software. The statistical significance between two groups was determined using Student’s -*t* test. Differences between more than two groups were assessed using one-way ANOVA followed by Tukey’s test. Significance was stablished when *P* < 0.05.

### Ethics statement

Experiments with mice were approved by the Animal Research Ethics Committee of the University of Barcelona. PBMCs were isolated from buffy coats provided by the “Banc de Sang i Teixits” of Barcelona. This transfer fulfils the regulations approved by the Ethics Committe of this Institution.

## Results and discussion

### Sat is autotransported and its passenger domain is released into the medium in EcN cultures

**Fig. 1:**
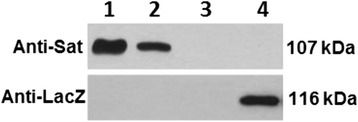
Western blot analysis of Sat secretion by EcN grown in LB. Several samples collected from an LB culture of EcN were processed and analysed with anti-Sat antibodies or with anti-LacZ antibodies as a control of cytosolic contamination: lane 1, TCA-precipitated cell-free supernatant before isolation of vesicles (total secreted proteins); lane 2, TCA-precipitated cell-free supernatant after removal of vesicles (soluble secreted proteins); lane 3, isolated OMVs; lane 4, cell extract (5 μg). IPTG (5 mM) was added to the culture medium to induce *lacZ* expression

Previous studies by our group identified Sat-specific peptides in the proteome of OMVs isolated from EcN cultures grown in LB [33]. To examine whether EcN Sat is secreted, supernatants of LB cultures were processed and analyzed by Western blot using antibodies anti-Sat obtained against a peptide of the passenger domain. Culture was centrifuged and the supernatant filtrated to eliminate bacteria. An aliquot of this cell-free supernatant was precipitated with TCA to obtain total secreted proteins. The remaining cell-free supernatant was centrifuged at 150,000 x *g* for 90 min to isolate OMVs from soluble secreted proteins. The presence of Sat in these fractions was analyzed by Western blot. A 107 kDa protein band was immunodetected in the total supernatant (Fig. 1, lane 1) and in the OMV free-supernatant (Fig. 1, lane 2), but not in the OMV fraction (Fig. 1, lane 3). To rule out cytosolic contamination due to cell lysis, immunoblots were probed with antibodies against the intracellular protein β-galactosidase. These results indicated that EcN Sat is secreted into the medium as a soluble 107-kDa protein. The C-terminal β-barrel domain of type V autotransporters remains inserted in the outer membrane. The absence of a cross-reactive band in the isolated OMVs is compatible with this secretion pathway, since the anti-Sat antibodies used do not recognize protein sequences of the β-barrel domain. The absence of a cross-reactive band in cellular extracts (Fig. 1, lane 4) is in accordance with the extracellular location of autotransporters of the SPATE family.

### The Sat protein encoded in the EcN genome displays serine protease activity

**Fig. 2:**
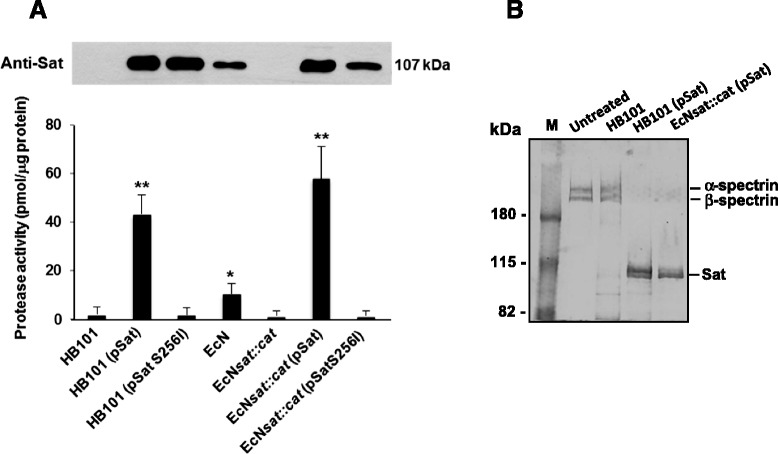
Serin protease activity of EcN Sat. **a** Concentrated culture supernatants (200 μg total protein) from the indicated *E. coli* strains were incubated with 1 mM methoxysuccynil-Ala-Ala-Pro-Val p-nitroanilide at 37 °C for 18 h. Activity values, expressed as pmoles/μg protein at18 h, were the mean ± SE of three independent assays. Asterisks indicate values significantly different from that of the knockout mutant EcN*sat::cm* (^*^ *P* < 0.05; ^**^ *P* = 0.000). Expression of Sat analysed by Western blot in each culture supernatant is shown in the upper panel. **b** Spectrin degradation by EcN Sat. Spectrin (4 μg) was incubated with the indicated culture supernatants or left untretaed. Reaction products were separated by SDS-6 % PAGE and stained by Commassie Blue. M, molecular mass marker

EcN Sat displays the functional motifs of the serine proteases of the SPATE family, including the catalytic triad formed by H, D and S residues, with the catalytic S256 residue comprised in the conserved GDSGSG motif (positions 254 to 259 in the protein sequence). To examine whether EcN Sat displays serine protease activity we cloned the EcN *sat* gene in pBR322 (pSat) and constructed the Sat mutant S256I by site-directed mutagenesis (pSat-S256I). These constructs were transformed into the *E. coli* strain HB101 lacking the *sat* gene, as well as into the mutant strain EcN*sat::cat* for comparison. Protease activity was assayed in concentrated culture supernatants obtained from LB cultures of these transformed cells by measuring hydrolysis of the colorimetric substrate methoxysuccinyl-Ala-Ala-Pro-Val p-nitroanilide. We selected this substrate based on published results on the activity and specificity of several proteases of the SPATE family [19]. Supernatants of strains EcN, HB101 and EcN*sat::cat* were processed in parallel. In all samples the presence of Sat was assessed by Western blot using anti-Sat specific antibodies. Results are presented in Fig. 2a. Supernatants of EcN wild type displayed proteolytic activity, which was abolished by disruption of the *sat* gene (EcN*sat::cat*). The proteolytic activity in supernatants of Sat deficient cells transformed with pSat confirmed that the *sat* gene of the probiotic strain EcN encodes a functional serine protease. Consistently, all enzymatically active samples were completely inhibited by 30 min pre-incubation with the serine protease inhibitor PMSF. Expression from the recombinant plasmid pSat yielded activity levels, which were between 5–7 times higher than the expression from the chromosomal gene in EcN strain. In these supernatants, there was a correlation between proteolytic activity values and the amount of Sat protein immunodetected with specific antibodies. The mutated Sat protein expressed from the recombinant plasmid pSat-S256I had negligible protease activity. These data confirmed Ser256 as the catalytic residue of EcN Sat.

To test whether the EcN toxin was active on known protein Sat substrates *in vitro*, concentrated culture supernatants of the recombinant HB101 or EcN*sat::cat* strains expressing the wild-type Sat protein were incubated with spectrin. Supernatants of HB101 were processed in parallel as a control. As shown in Fig. 2b, EcN Sat is able to degrade spectrin subunits. These results indicated that Sat from the probiotic strain is secreted as an active serine protease as the toxin from pathogenic *E. coli* strains.

### Analysis of EcN *sat* expression during colonization of the mouse intestine

Since the ecological niche of the commensal strain EcN is the intestinal tract we sought to analyze whether Sat is expressed *in vivo* during intestinal colonization of mice. To monitor *sat* expression, the *sat* promoter of strain EcN was cloned upstream the reporter gene *gfpmut3.1* of plasmid pFU34. This fragment extended 349 bp upstream from the ATG start codon. The recombinant plasmid pFU34-sat was transformed into EcN cells and expression of green fluorescent protein (GFP) was visualized by fluorescence microscopy. To check the functionality of this construct we first analyzed GFP expression in cells grown to exponential phase in LB as well as in DMEM or glucose minimal medium. To analyze whether expression of *sat* is temperature-regulated, cultures were incubated at either 37 °C or 25 °C. EcN cells harboring the vector pFU34 were processed in parallel for comparison. Results showed expression from the *sat* promoter in all growth conditions tested (see Additional file 1: Figure S1). Many virulence genes of bacterial pathogens that infect humans are controlled by temperature and their expression is induced at 37 °C [37–39]. Our results rule out temperature regulation of *sa*t expression in EcN. To quantify the level of GFP expression in the culture media used, the emitted fluorescence was measured using the Turner BioSystems Modulus™ II Microplate reader. Control cultures of EcN harboring the vector pFU34 were performed in parallel. Expression values from the *sat* promoter were 2.4x10^4^ RFU/OD for cells grown in LB, 2.6x10^3^ RFU/OD in MM-glucose and 2.9x10^3^ RFU/OD in DMEM. Thus, maximal expression of *sat* was achieved in LB. Addition of compounds present in the gut such as sodium bicarbonate (3.7 g/l) or sodium deoxycolate (0.5 mg/ml) to cultures of EcN (pFU34-sat) in LB or MM-glucose did not increase the level of GFP expression from the *sat* promoter.

The absence of fluorescence background in control cells harboring the vector pFU34 grown in LB (see Additional file 1: Figure S1) prompted us to use this high-copy number fusion system for the analysis of *sat* expression *in vivo*. To this end, we inoculated orogastrically 8- to 10-week old CD-1 male mice with a suspension of EcN cells harboring plasmid PFU34-sat (*n* = 2) or EcN cells harboring vector PFU34 (*n* = 2). Control mice (*n* = 2) were given PBS. Before starting the experiment stools were collected to assess the absence of Ap-resistant resident microbiota. Mice were given water containing 2 g/l ampicillin *ad libitum* 24 hours prior to inoculation and through the experiment. Five days post-inoculation, fresh fecal samples, as well as the mucus layer from ileon and colon sections, were collected and processed for quantification of bacteria by growth on LB-Ap plates. Two colonies of each bacterial count were analyzed by PCR with *sat* specific primers to confirm that the estimated colonization rates corresponded to the inoculated bacteria. A drop of each fecal or mucosal suspension was deposited on a slide and visualized by fluorescence microscopy.

**Fig. 3:**
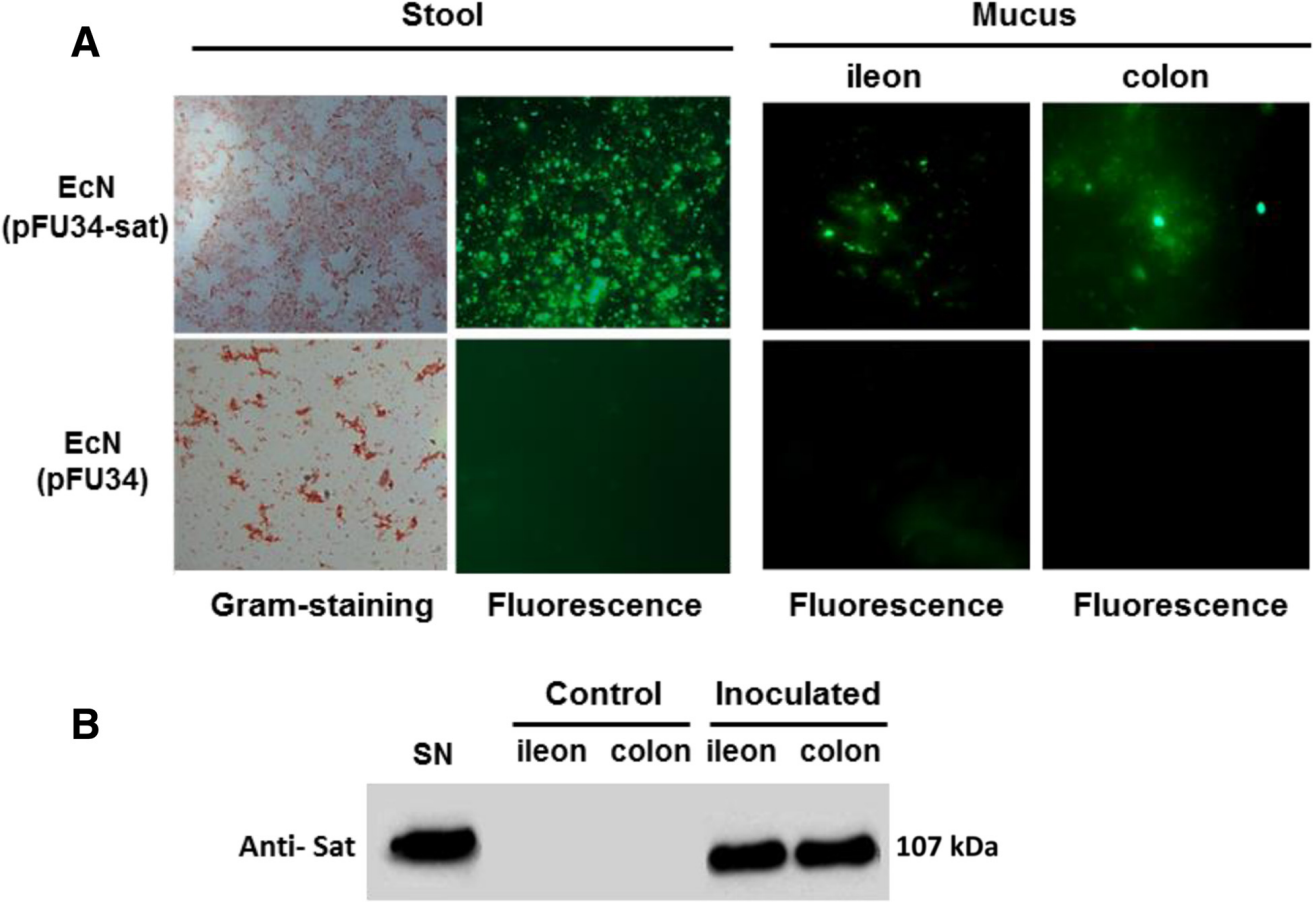
Analysis of Sat expression during EcN colonization of mouse intestine. **a** *In vivo* expression of the promoter fusion *sat-gfp*_*mut3.*1_ in samples derived from the intestine of mice colonized with EcN harbouring pFU34-sat, or pFU34 as a control. Doses of 2x10^5^ bacteria of the indicated strains were used to inoculate orogastrically CD-1 mice (eight- to ten-weeks old), which were provided with drinking water containing ampicillin from 48 hours prior to inoculation and over the entire course of the experiment. Five days post-inoculation fresh stool samples, as well as the mucus layer from the ileon and colon sections, were collected. A drop of each fecal or mucosal suspension was deposited on a slide and visualized by fluorescence microscopy. Representative images derived from one mouse of each type are shown. **b** Western blot analysis of EcN Sat adhered to intestinal mucus of colonized mice. Ileon and colon mucus collected from inoculated mice were homogenized and proteins precipitated with TCA. Equivalent samples collected from non-inoculated mice were processed in parallel for control. Cell-free supernatants (SN) from EcN cultures grown in LB were used as a positive immunoblotting control

The level of colonization was similar for all inoculated mice, around 1-2x10^9^ CFU/g of stool and 3-5x10^9^ CFU/g of mucus in ileon and colon sections (see Additional file 2: Table S1) whereas samples from non-inoculated mice gave no bacterial counts on LB-Ap plates. Expression of GFP was visualized only in samples collected from the two mice colonized with EcN harbouring the fusion plasmid pFU34-sat (Fig. 3a). These results indicated that EcN *sat* is expressed in distinct niches in the mouse intestine.

Expression of chromosome-encoded Sat was analysed by Western blot analysis of intestinal mucosa samples collected from inoculated mice using anti-Sat antibodies. Equivalent samples from non-inoculated mice were processed in parallel as a control. The presence of a 107 kDa protein in colon and ileon samples from inoculated mice confirmed *sat* expression during EcN intestinal colonization (Fig. 3b).

### Sat-mediated cytotoxic effects on undifferentiated epithelial cells

The Sat protein secreted by UPEC and DAEC strains exerts cytotoxic effects on several epithelial cell lines. Internalized Sat induces disruption of F-actin cytoskeleton, vacuolization and cell death in non-polarized epithelial cells [22], but these effects are not observed in fully differentiated Caco-2 cells [23]. As EcN Sat is an active protease we sought to examine whether the protein encoded in this probiotic strain could induce cytotoxicity in sub-confluent HeLa cells. To avoid cross-effects of other putative SPATEs encoded in the EcN genome like Vat [12], cell-free supernatants of strain HB101 expressing EcN Sat were used for this study. A decrease of cell viability, measured by the MTT assay, was observed in HeLa cells from 8 h-treatment with Sat-containing concentrated culture supernatants (see Additional file 3: Figure S2).

### Effect of EcN Sat on paracellular permeability of Caco-2 cell monolayers

**Fig. 4:**
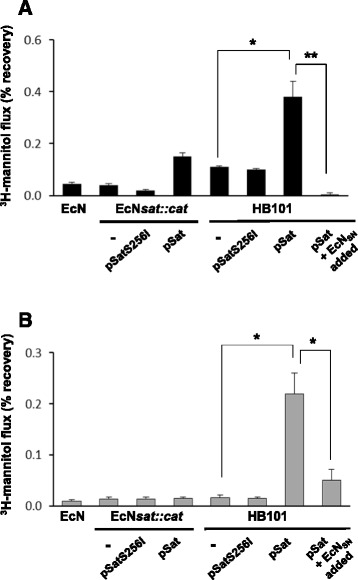
Effect of EcN Sat on the paracellular permeability to ^3^H-mannitol in Caco-2 cell monolayers. Paracellular passage of [^3^H]-D-mannitol from apical to basolateral compartments of Caco-2 monolayers after 3-h incubation with suspensions of the indicated bacteria (MOI 100) (**a**) or with the corresponding concentrated culture supernatants (200 μg) (**b**). Equivalent amounts of recombinant Sat were assessed by Western blot. The last bar of each panel corresponds to cells incubated with the recombinant HB101(pSat) samples in the presence of EcN culture supernatant (indicated as EcN_SN_ added). Data are presented as the percentage of tracer recovery relative to the total tracer used. Values were corrected subtracting the recovery of control cells incubated with the medium. Asterisks indicate values significantly different from that of EcN*sat::cm* (^*^ *P* < 0.05; ** < 0.01)

To test whether Sat from the probiotic strain EcN affects the intestinal epithelial barrier, paracellular permeability of polarized Caco-2 cells was measured using ^3^H-mannitol as a marker at 3 h post-infection with wild-type EcN and the recombinant strains HB101 and EcN*sat::cat* expressing Sat from plasmid pSat, or expressing the inactive Sat-S256I protein (Fig. 4a). We assessed that both recombinants strains (HB101 and EcN*sat::cat*) displayed equal growth rates in the culture medium DMEM. Incubations with the corresponding concentrated cell-free supernatants were performed in parallel (Fig. 4b). A significant increase in the paracellular passage of mannitol from apical to basolateral compartment was observed only in Caco-2 monolayers incubated with samples of HB101 bearing plasmid pSat (either bacterial suspensions or concentrated cell-free supernatants). Expression of the inactive Sat-S256I variant in this strain did not affect paracellular permeability, which indicates that activity of EcN Sat on the barrier integrity depends on its proteolytic activity. These results are in accordance with those described for the Sat protein from DAEC strains [23] and suggest that the Sat protein encoded by probiotic strains may also induce disassembly of tight junction proteins. However, expression of comparable EcN Sat levels in the same probiotic background (strain EcN*sat::cat* bearing pSat) did not affect the paracellular permeability to ^3^H-mannitol (Fig. 4). It is known that EcN positively modulates the epithelial barrier function by promoting the formation of tight junctions [5–7]. Therefore, other factors secreted by this probiotic strain may counteract the negative effect of Sat on tight junctions-associated proteins. To test this hypothesis Caco-2 monolayers were incubated either with bacterial suspensions or concentrated culture supernatants of strain HB101(pSat) in the presence of cell-free supernatants collected from the probiotic strain. In both cases, supernatants from the probiotic strain confer protection against the Sat effect on paracellular permeability to mannitol (Fig. 4). Cell free supernatants obtained from the mutant strain EcN*sat::cat* produced the same protective effect (not shown).

**Fig. 5:**
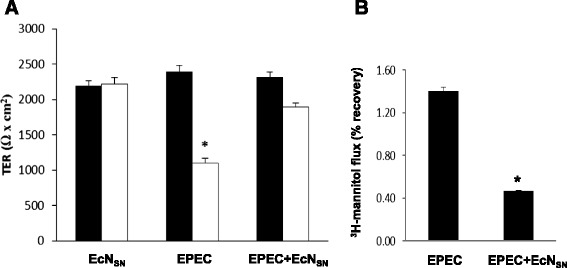
EcN culture supernatants impair EPEC-mediated effects on epithelial cell barrier. Caco-2 monolayers grown on Transwell filters were incubated with EPEC alone (MOI 50) or with EPEC (MOI 50) in the presence of EcN cell-free culture supernatant (200 μg total protein). Incubation of Caco-2 cells with EcN supernatants (EC_SN_) were performed in parallel as a control. **a** TER values before (black bars) and after 2 hours incubation (white bars) were presented. **b** Paracellular passage of [^3^H]-D-mannitol from apical to basolateral compartments of Caco-2 monolayers after 2-h incubation. Data are expressed as the percentage of tracer recovery relative to the total tracer used (^*^ *P* < 0.05;)

It has been described that the TcpC protein of EcN promotes upregulation of claudin-14 in HT-29/B6 cells, resulting in increased TER and reduced paracellular permeability to mannitol [7]. Other studies aimed at deciphering the mechanism by which EcN restores barrier integrity after enteropathogenic E. coli (EPEC) damage revealed that this probiotic strain induces ZO-2 expression and its redistribution to the cell membrane in polarized T84 cells. In this case the bacterial factor responsible for this effect was not identified [6]. Impairment of Sat-mediated damage of Caco-2 cell barrier by concentrated EcN culture supernatants (Fig. 4) indicated that the protective effect could be attributed to a secreted factor. Thus, we sought to evaluate the ability of EcN supernatants to prevent barrier disruption by EPEC in polarized Caco-2 cell monolayers. The reduction in TER caused by EPEC after 2 h-incubation was almost completely abolished when infection was performed in the presence of concentrated EcN supernatants (Fig. 5a). In addition, the EPEC- deleterious effect on paracellular permeability to mannitol was reduced by 70 % in the presence of EcN supernatant (Fig. 5b).

Overall these results indicate that EcN Sat is able to alter the paracellular permeability of Caco-2 cell monolayers. However, other factors secreted by the probiotic strain prevent barrier disruption caused by pathogen-related factors.

### Effect of EcN Sat on gut colonization in mice

Some serine proteases of the SPATE family such as Pic [35] or SigA [40] contribute to intestinal colonization in animal models. In pathogenic *E. coli* strains, the corresponding knockout mutants displayed reduced colonization levels when compared to the wild- type strain. In contrast, other proteases of this family like SepA are not required for intestinal colonization [40].

**Fig. 6:**
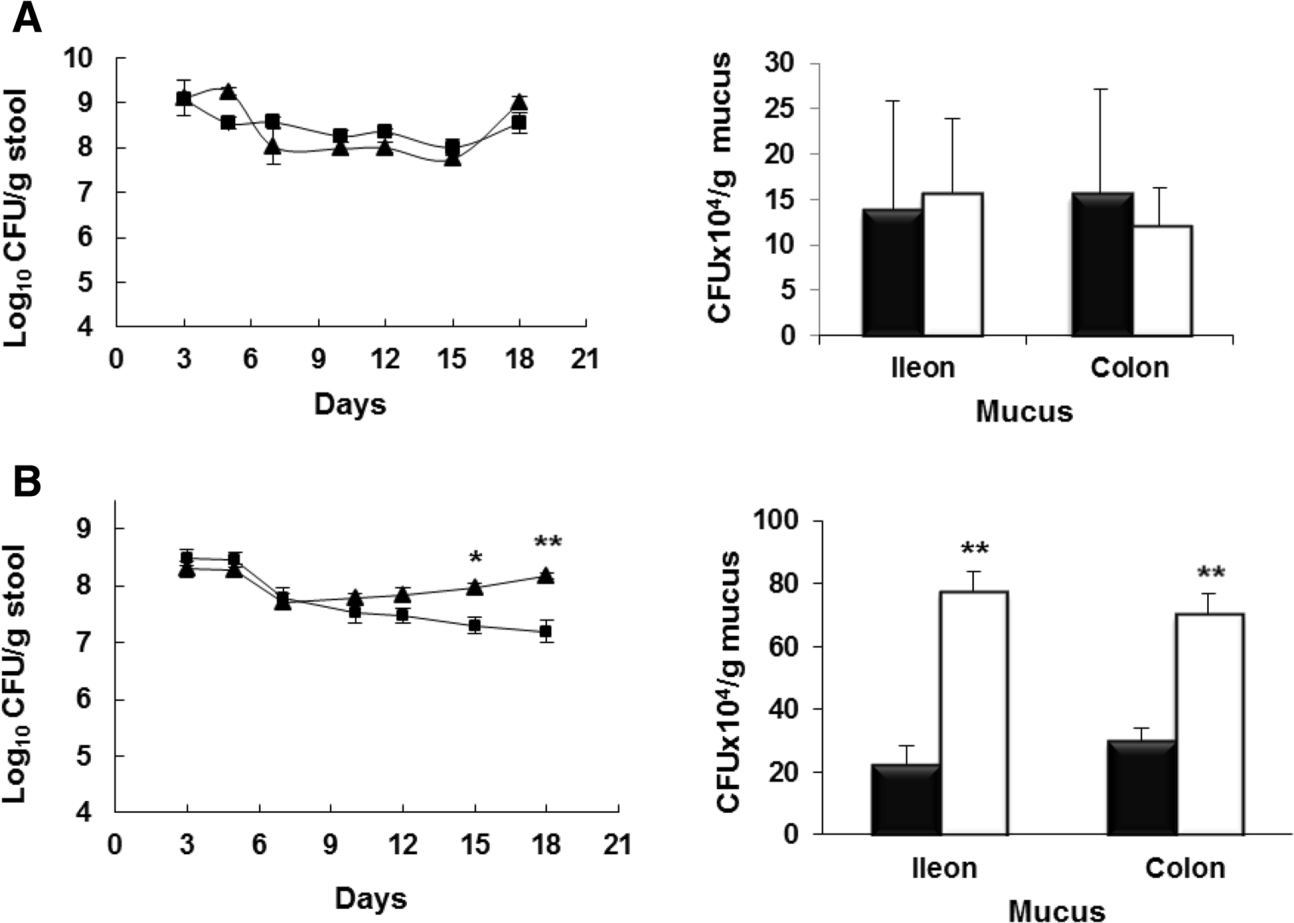
Colonization of the mouse intestine by EcN and EcN*sat::cat*. **a** Two sets of four mice were fed independently with 2x10^5^ CFU of strain EcN Sm^r^ Rf^r^ (squares) or the mutant strain EcN*sat::cat* Sm^r^ Rf^r^ Cm^r^ (triangles). **b** Competition experiments: One set of four mice were fed with 2x10^5^ CFU of a mixture (1:1) of strains EcN Sm^r^ Rf^r^ (squares) and the mutant strain EcN*sat::cat* Sm^r^ Rf^r^ Cm^r^ (triangles). Three independent biological assays were performed for these competition experiments (total *n* = 12). At the indicated times, stool samples were collected and processed for bacterial counting. Results are expressed as Log_10_ CFU/g stool (left panels). At day 18, mice were sacrificed and sections from ileum and colon were removed from each mouse. The mucus layer from each fragment was scraped, homogenized and processed for bacterial quantification. Results, expressed as CFUx10^4^/g of mucus, were the mean ± SE of the three independent biological assays (right panels). Asterisks indicate values significantly different from that of the parental strain EcN (*p < 0.05, **p = 0.000)

To examine whether Sat has a role in gut colonization, the streptomycin-treated mouse model was used to compare the colonization capacity of the Sat deficient mutant versus the wild-type strain. Mice were inoculated with strain EcN Sm^r^ Rf^r^ (*sat* wild-type group) or with EcN*sat::cat* Sm^r^ Rf^r^ Cm^r^ (*sat* mutant group). No significant differences in stool bacterial counts were observed between the two groups through the experiment. For both strains colonization levels were between 10^8^ to 10^9^ CFU/g of stool (Fig. 6a). Quantification of adhered bacteria to mucus samples collected from ileon and colon on the last day of the experiment also yielded no significant differences between these strains when tested independently (Fig. 6a, right panel). These results suggested that Sat is not required for EcN colonization of the mouse intestine. They are in accordance with results obtained with UPEC strains in murine models of ascending urinary tract infections, in which the *sat* mutant colonized bladder and kidneys to the same extent as the wild type strain [18].

To better assess the contribution of Sat to intestinal colonization we approached competition experiments between the specific *sat* mutant and the wild-type strain. This approach is more sensitive for the evaluation of the colonization efficiency in streptomycin-treated mice [35]. Mice were given simultaneously 1x10^5^ CFU of each strain (co-infection group), and bacteria in stools were counted for 18 days. Results showed that wild type EcN was outcompeted by the *sat* mutant strain (Fig. 6b). The values of CFU/g stool were similar for both strains within the first several days (around 5x10^8^ CFU/g of stool) but significant differences were observed around day 10. After this time, colonization by wild-type EcN fell to 10^7^ CFU/g stool at day 18. At the end of the experiment, bacterial counts in stools yielded percentages of 70 % for the mutant strain EcN*sat::cat* versus 30 % for the wild-type EcN. At day 18, quantification of adhered bacteria to the intestinal mucus layer also showed a higher proportion of the Sat deficient strain (70–80 % versus 20-30 % of the wild type strain).

Recently, an *in vivo* study performed with *Citrobacter rodentium* assigned to a class I SPATE protein (Crc1) a putative role in immunomodulation by controlling leukocyte infiltration and proinflammatory cytokines levels in mouse intestine [41]. Moreover, some class II SPATE proteins, like Pic and Tsh, cleave leukocyte surface glycoproteins causing diverse effects in leukocyte activation, migration and signalling [42]. A proteomic analysis aimed at identifying potential host targets of Sat from UPEC revealed a protein in the kidney membrane that is similar to the leukocyte function associated molecule 1 (LFA-1) [20]. LFA-1 is a member of the β2-integrin family of cell surface receptors. This protein is a multifunctional adhesion molecule involved in various interactions in the immune system being involved in leukocyte migration [43]. Since lymphocyte migration to the gut is required for immune homoeostasis, we sought to analyse whether surface associated LFA-1 in leukocytes could be degraded by EcN Sat. To this end we performed flow cytometry analyses of human PBMCs treated for 3 h with supernatants of strains EcN*sat::cat*, EcN*sat::cat* bearing plasmid pSat, HB101, and HB101 bearing either plasmid pSat or pSat-S256I. Labelling with anti-LFA-1 (anti CD-11/CD-18) antibodies that recognize the native protein followed by Alexa Fluor 488 goat anti-mouse IgG did not show any significant difference between samples. These results indicated that leukocyte-associated LFA-1 is not a target of EcN Sat. Therefore the ability of the *sat* mutant strain to outcompete EcN in the intestinal tract cannot be attributed to a Sat effect on gut immune function mediated by LFA-1.

At present we cannot explain why the *sat* mutant outcompetes wild type EcN in the streptomycin-treated mouse model. Since growth rates of both strains in *in vitro* cultures are indistinguishable, this fact could be associated to a better performance of the *sat* mutant in the mouse intestine. In this sense, it has been described that mutations in *envZ*, which result in reduced expression of some outer membrane proteins, render EcN a better colonizer than the wild type EcN [44]. As EcN resides associated with other members of the microbiota in the mouse intestine, authors hypothesize that surface differences between the *envZ* mutant and wild-type EcN could result in different binding affinities for mixed biofilms, allowing the mutant strain to colonize niches than the wild-type strain cannot do. These mutants display reduced levels of specific porins, being more resistant to colicin and bile salts. In this context, the outer membrane of the *sat* mutant lacks the autotransporter β-barrel domain, which presents a porin structure. We may speculate that this difference in bacterial surface may modify interactions with other microbiota members and/or the sensitivity to antimicrobial products released by other intestinal strains.

### Prevalence of the *sat* gene in natural *E. coli* intestinal isolates

Epidemiological studies have shown a high prevalence of the *sat* gene in *E. coli* strains associated with urinary tract infections [18, 23]. Concerning strains causing intestinal infection, *sat* was detected in 46 % of 35 Afa/Dr DAEC strains isolated from diarrhoeagenic samples [23]. Similarly, a study aimed at evaluating the prevalence of several cytotoxins of the SPATE family in enteroaggregative *E. coli* (EAEC) isolates showed high frequency of the *sat* gene in this group (74.5 % of 55 strains analysed). However, this study also revealed the absence of *sat* in all the enterotoxigenic *E. coli* (ETEC), EPEC or enteroinvasive *E. coli* (EIEC) isolates analysed in parallel (10 strains of each group) [45]. In order to assess the prevalence of *sat* among natural *E. coli* intestinal isolates we performed a PCR survey. For this study we selected 29 *E. coli* strains of the ECOR collection [46] recovered from stools of healthy individuals. The strains were examined by PCR using specific primers that allow amplification of the DNA sequence encoding Sat. This screening showed ten positive isolates that yielded the expected *sat* amplification fragment (34.8 %). Accuracy of the PCR product was verified by nucleotide sequencing. Distribution of these ten *sat* positive isolates among the *E. coli* phylogenetic groups revealed a high prevalence of strains belonging to phylogenetic groups associated with virulent strains that cause extra-intestinal infections (80 %), namely group D (6 isolates) and group B2 (2 isolates) (Table 2). Only one intestinal isolate fits into the phylogenetic group A generally associated with non-pathogenic *E. coli* strains [47]. Expression of *sat* in these strains was evaluated by immunodetection of the secreted protein with specific antibodies against the passenger domain of Sat. All the intestinal isolates with the *sat* gene yielded a cross-reactive protein band in the Western blot analysis.

**Table 2:** Characteristics of the *sat* positive strains from the ECOR collection isolated from stools of healthy humans^a^

			Sat amino acid residue at position		
Strain	Serotype	Group	352	729	894
CFT073^b^	O6:K2:H1	B2	D	D	N
IH11128^b^	O75:K5:H^−^	B2	D	D	N
EcN^b^	O6:K5:H1	B2	N	N	D
ECOR24	O15:NM	A	N	N	D
ECOR35	O1:NM	D	N	N	D
ECOR36	O79:H25	D	N	N	D
ECOR38	O7:NM	D	N	N	D
ECOR39	O7:NM	D	N	S	D
ECOR41	O7:NM	D	N	S	D
ECOR43	ONT:HNT	E	N	S	D
ECOR49	O2:NM	D	N	N	D
ECOR51	O25:N	B2	N	N	D
ECOR56	O6:H1	B2	D	D	N

^a^ Analyzed strains that did not yield *sat* amplification in the PCR screening: ECOR strains 1, 2, 4, 5, 6, 8, 9, 10, 12, 13, 15, 26, 28, 42, 53, 54, 56, 59, 61 and 63

^b^ Strains CFT073 (UPEC), IH11128 (Afa/Dr DAEC) and EcN were included for comparison

The Sat protein from the probiotic EcN differs in eight residues with respect to the UPEC protein, whereas only three changes that fit into the non-conservative category (N352D, N729D and D894N) were observed when compared to the protein from DAEC strains (see Additional file 2: Table S2). Interestingly, among the commensal *sat* positive strains identified in this study, only EcoR56 displayed the same amino acid sequence as the Sat protein from pathogenic DAEC (D352, D729 and N894). The other strains expressed a Sat protein with N352, N729 and D894 residues as EcN Sat or with only one conservative change (N729S) (Table 2).

The presence of multiple virulence factors in the probiotic strain EcN raises the question as to why this strain does not cause symptomatic infections. Several possibilities have been proposed [12]. First, virulence genes may be inactivated in probiotic or commensal strains. On the other hand, some of these genes may encode factors that increase overall fitness during colonization of the human gut. As stated above, the distinction between virulence and fitness factors is not always clear. In addition, the role of a given virulence factor in pathogenesis may depend on the genetic background of the strain. In this sense, TcpC protein that is expressed by the probiotic strain EcN has been shown to contribute to UPEC virulence. This protein, with high prevalence in Gram-negative pathogens, contains a Toll/IL-1 receptor (TIR) -binding domain. In pathogens, TcpC interacts with Myd88 and inhibits TLR signalling and downstream pathways. By this mechanism, TcpC impairs innate immunity causing inflammation and tissue damage [48, 49]. However, in strain EcN, which displays anti-inflammatory and immunomodulatory effects, TcpC was shown to improve the intestinal barrier through upregulation of the tight junction-protein claudin-14 [7].

## Conclusions

Sat is one of the proteins with cytotoxic activity encoded in the EcN genome. Here we show that EcN Sat is a functional protease that is expressed in the mouse intestinal tract. Our results, together with other reports, indicate that both Sat function and Sat cytotoxicity may rely on other host- and bacterial factors. Regarding host factors, it should to be stressed that Sat cytotoxic effects depend on the cellular line and the differentiation state. Sat-mediated disruption of actin cytoskeleton, vacuolization and cell detachment were not observed in monolayers of polarized Caco-2 cells, a model that mimics intestinal epithelium and barrier. It has been suggested that putative regulatory mechanisms may operate in polarized epithelia, which may impair Sat access or action on target intracellular proteins [23]. Considering the strain background, our results show that Sat-mediated increase in paracellular permeability of Caco-2 monolayers is impaired by other secreted factors encoded in the probiotic strain EcN. Thus, the role of Sat in intestinal pathogenesis relies on the pattern of genetic determinants responsible for the bacterial pathotype. Expression of particular virulence factors may help Sat to meet specific intracellular target substrates.

At present the role of Sat in the probiotic strain remains elusive. Although Sat contributes to pathogenicity in the urine tract, the prevalence of *sat* in *E. coli* strains isolated from intestinal microbiota of healthy individuals suggests that Sat may not act as a virulence factor in the human gut. This hypothesis is in accordance with the very low prevalence of *sat* among several *E. coli* enteropathogenic groups (ETEC, EIEC, EPEC). The high prevalence of *sat* in intestinal *E. coli* strains of group D suggests that the presence of this gene may be advantageous for pathogenesis in extra-intestinal environments.

## Additional files

Additional file 1: Figure S1. Expression analysis of the transcriptional fusion *sat-gfp*_*mut3.1*_ in EcN grown in diferent conditions. (PDF 173 kb) Additional file 2: Table S1. Bacterial populations in mouse stool, and ileal and colonic mucus at five days postinoculation with the indicated strains. Table S2. Variations in Sat sequence among *E.coli* strains. (PDF 165 kb) Additional file 3: Figure S2. Effect of EcN Sat on cell viability. (PDF 197 kb)

## Abbreviations

Ap: Ampicillin
CFU: Colony forming units
Cm: Chloramphenicol
DAEC: Diffusely adherent *Escherichia coli*
DMEM: Dulbecco’s modified Eagle medium
EAEC: Enteroaggregative *Escherichia coli*
EcN: *Escherichia coli* Nissle 1917
EIEC: Enteroinvasive *Escherichia coli*
EPEC: Enteropathogenic *Escherichia coli*
ETEC: Enterotoxigenic *Escherichia coli*
FCS: Fetal calf serum
GFP: Green fluorescent protein
IPTG: Isopropyl-β-D-1-thiogalactopyranoside
LB: Luria-Bertani broth
LFA-1: Leukocyte function associated molecule
MM: Minimal medium
MOI: Multiplicity of infection
OD: Optical density
OMVs: Outer membrane vesicles
PAGE: Polyacrylamide gel electrophoresis
PBMCs: Peripheral blood mononuclear cells
PBS: Phosphate buffer saline
PMSF: Phenylmethanesulfonyl fluoride
Rf: Rifampicin
RFU: Relative fluorescence units
Sat: Secreted autotransporter toxin
SDS: Sodium dodecyl sulphate
Sm: Streptomycin
SPATEs: Serine protease autotransporters of *Enterobacteriaceae*
Tc: Tetracycline
TCA: Trichloroacetic acid
TER: Transepithelial electrical resistance
UPEC: Uropathogenic *Escherichia coli*
