# Supplementary material for: The secreted autotransporter toxin (Sat) does not act as a virulence factor in the probiotic Escherichia coli strain Nissle 1917
Source: BMC Microbiol. 2015 Oct 30;15:250. doi: 10.1186/s12866-015-0591-5 (PMC4628265; doi:10.1186/s12866-015-0591-5)
Supplement: Additional file 1: Figure S1. — Expression analysis of the transcriptional fusion sat-gfp mut3.1 in EcN grown in diferent conditions. (PDF 173 kb) [file 12866_2015_591_MOESM1_ESM.pdf]

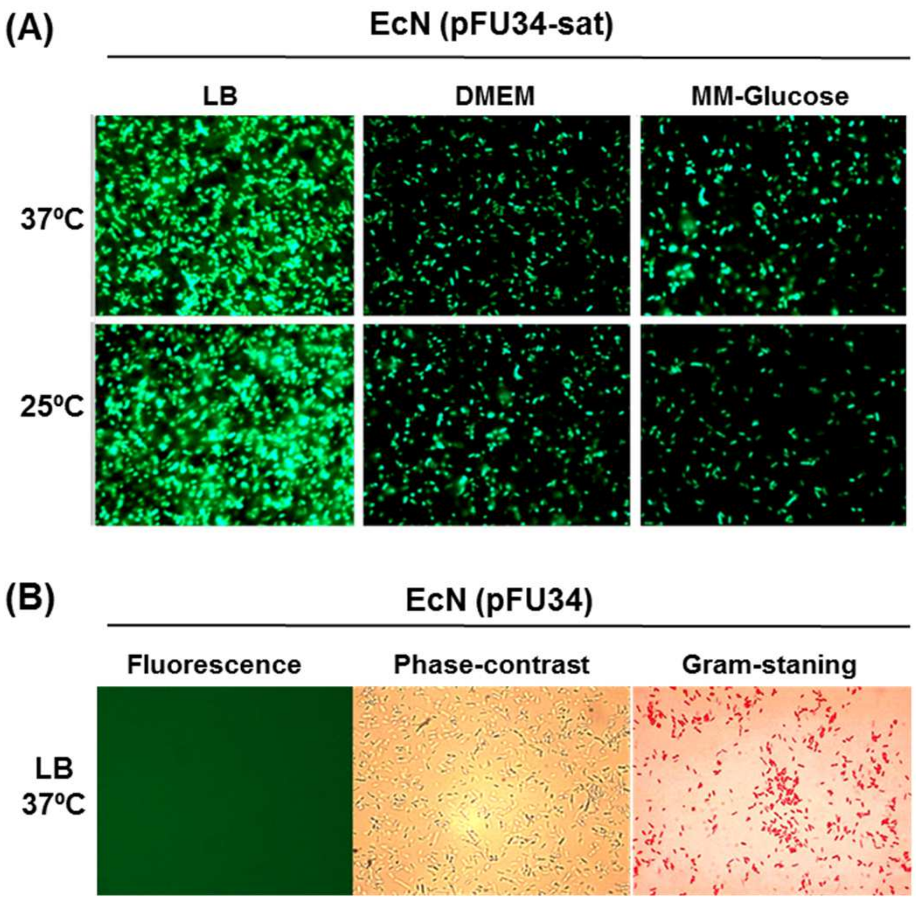

Figure S1. Expression analysis of the transcriptional fusion *sat-gfp*$_{mut3.1}$ in EcN grown in different conditions. EcN cells harbouring pFU34-sat were diluted 1:50 in the indicated culture media from overnight cultures and grown to late exponential phase at 37ºC or 25ºC. Bacteria were detected by expression of *sat-gfp*$_{mut3.1}$ using a fluorescence microscopy (GFP channel). LB cultures of EcN strain harbouring the vector pFU34 were processed in parallel as a control. In this case bacteria were visualized by phase-contrast and Gram-staining.
