# Supplementary material for: The secreted autotransporter toxin (Sat) does not act as a virulence factor in the probiotic Escherichia coli strain Nissle 1917
Source: BMC Microbiol. 2015 Oct 30;15:250. doi: 10.1186/s12866-015-0591-5 (PMC4628265; doi:10.1186/s12866-015-0591-5)
Supplement: Additional file 2: Table S1. — Bacterial populations in mouse stool, and ileal and colonic mucus at five days postinoculation with the indicated strains. Table S2. Variations in Sat sequence among E.coli strains. (PDF 165 kb) [file 12866_2015_591_MOESM2_ESM.pdf]

Table S1. Bacterial populations in mouse stool, and ileal and colonic mucus at five days postinoculation with the indicated strains

Inoculated strain	CFU/g stool	Ileon CFU/g mucus	Colon CFU/g mucus
EcN (PFU34-sat)	$1.4x10^{9}$	$3.4x10^{4}$	$4.1x10^{4}$
EcN (PFU34-sat)	$1.2x10^{9}$	$4.1x10^{4}$	$3.9x10^{4}$
EcN (pFU34)	$1.1x10^{9}$	$2.4x10^{4}$	$3.0x10^{4}$
EcN (pFU34)	$1.6x10^{9}$	$5.0x10^{4}$	$3.5x10^{4}$

Table S2. Variations in Sat sequence among *E.coli* strains

	UPEC	DAEC	Probiotic *E. coli*	
AA position	CFT073	IH11128	EcN	ABU 83972
140	R	K	K	K
352	D	D	N	N
579	S	T	T	T
612	Y	H	H	H
669	V	A	A	A
729	D	D	N	N
894	N	N	D	D
1041	I	M	M	M
