# Supplementary material for: The secreted autotransporter toxin (Sat) does not act as a virulence factor in the probiotic Escherichia coli strain Nissle 1917
Source: BMC Microbiol. 2015 Oct 30;15:250. doi: 10.1186/s12866-015-0591-5 (PMC4628265; doi:10.1186/s12866-015-0591-5)
Supplement: Additional file 3: Figure S2. — Effect of EcN Sat on cell viability. (PDF 197 kb) [file 12866_2015_591_MOESM3_ESM.pdf]

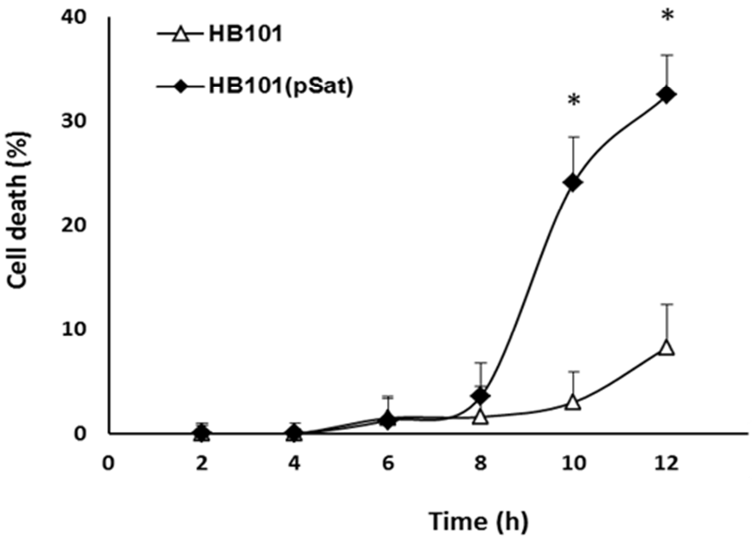

Figure S2. Effect of EcN Sat on cell viability. Cell viability was determined by MTT assay in HeLa cells treated with concentrated cell-free supernatants (200 μg) of recombinant HB101(pSat) or the parental HB101 strain. *P<0.01 compared with non-tretaed control cells.
